# Accuracy Improvement for Predicting Parkinson’s Disease Progression

**DOI:** 10.1038/srep34181

**Published:** 2016-09-30

**Authors:** Mehrbakhsh Nilashi, Othman Ibrahim, Ali Ahani

**Affiliations:** 1 Department of Computer Science and Information Systems, Faculty of Computing, Johor, 81310 Skudai, Malaysia; 2 Department of Computer, Lahijan Branch, Islamic Azad University, Lahijan, Iran

## Abstract

Parkinson’s disease (PD) is a member of a larger group of neuromotor diseases marked by the progressive death of dopamineproducing cells in the brain. Providing computational tools for Parkinson disease using a set of data that contains medical information is very desirable for alleviating the symptoms that can help the amount of people who want to discover the risk of disease at an early stage. This paper proposes a new hybrid intelligent system for the prediction of PD progression using noise removal, clustering and prediction methods. Principal Component Analysis (PCA) and Expectation Maximization (EM) are respectively employed to address the multi-collinearity problems in the experimental datasets and clustering the data. We then apply Adaptive Neuro-Fuzzy Inference System (ANFIS) and Support Vector Regression (SVR) for prediction of PD progression. Experimental results on public Parkinson’s datasets show that the proposed method remarkably improves the accuracy of prediction of PD progression. The hybrid intelligent system can assist medical practitioners in the healthcare practice for early detection of Parkinson disease.

Parkinson’s Disease (PD) is a degenerative neurological disorder marked by decreased dopamine levels in the brain^1^,^2^,^3^. PD is the second most common neurodegenerative disorder following Alzheimer’s disease^4^,^5^,^6^,^7^,^8^. This disease is primarily characterized by four symptoms: tremor, rigidity, bradykinesia, and postural instability^9^,^10^. Though these symptoms present in varying degrees and combinations for different individuals, they are chronic and degenerative, progressively worsening over time. PD causes motor symptoms and non-motor symptoms that can affect the quality of daily life^3^,^11^. It has been shown that approximately 90% of the patients with PD show vocal impairment that includes impairment in the normal production of vocal sounds, which is dysphonia^12^,^13^. Recent studies have also shown that there is an association between the cumulative number of risk alleles and the risk of having PD^10^,^14^. The disease has influenced about 1–2% of people in the worldwide over 60 years of age^15^.

From the literature^16^,^17^,^18^, it has been emphasized that the main medical challenge is to correctly recognize the PD affected subjects at the early stage. The early diagnosis can assist the patients improve and maintain their quality of life^19^. However, due to symptom overlap with other diseases PD may be difficult to diagnose accurately, especially at the early stages of the illness^20^. In addition, traditional diagnosis of PD involves a clinician taking a neurological history of the patient and observing motor skills in various situations. Since there is no definitive laboratory test to diagnose PD, diagnosis is often difficult, particularly in the early stages when motor effects are not yet severe. Monitoring progression of the disease over time requires repeated clinic visits by the patient. There is no cure, but pharmacological treatment to manage the condition includes dopaminergic drugs.

Diagnosis is clearly a difficulty in PD management, and an effective screening process, particularly one that doesn’t require a clinic visit, would be beneficial^21^. Since PD patients exhibit characteristic vocal features, voice recordings are a useful and noninvasive tool for diagnosis. Thus, speech tests can be used for monitoring PD, due to vocal impairment being a common symptom and early indicator. Using an at-home recording device, such as one developed by Intel for PD telemonitoring, can conveniently allow PD patients’ health to be monitored remotely. If machine learning algorithms could be applied to a voice recording dataset to accurately diagnosis PD, this would be an effective screening step prior to an appointment with a clinician. Specified voice recordings can be passed through signal processing algorithms and a classification and regression method to predict a rating on the Unified Parkinson’s Disease Rating Scale (UPDRS)^19^. UPDRS, which displays presence and severity of symptoms, has been as the most widely used standardized scale for assessing Parkinsonism for quantifying the degree of impairment caused by Parkinsonian symptoms^8^,^9^,^22^.

Improving the predictive accuracy of PD progression has been an important task and an eye-catching topic these days^23^,^24^,^25^. A successful machine learning approach to accurately predict diseases from the real data would be applicable to many types of medical diagnosis. Accordingly, analysis of real datasets in clinical context by using machine learning and data mining techniques, methods, and tools assists to develop intelligent and knowledge based systems that can help clinicians in decision making^26^. There is a vast sea of different techniques and algorithms used in data mining especially for supervised machine learning techniques; therefore, selecting the appropriate techniques has been a challenge among researchers in developing the PD diagnosis systems. Classification and prediction methods have been successfully applied for many biological classification problems. The unsupervised learning is defined as cluster analysis. Clustering is a process of putting a set of observations into several reasonable groups according to certain measure of similarity within each group. Clustering methods have helped the diseases diagnosis systems in improving their predictive accuracy^27^,^28^,^29^. In this study, we take the advantages of clustering and prediction methods in order to improve predictive accuracy of PD progression. Accordingly, a new model is proposed with combination of prediction and clustering methods for predicting PD progression. We also use a noise removal method for dimensionality reduction of data. We apply Adaptive Neuro-Fuzzy Inference System (ANFIS) and Support Vector Regression (SVR) for prediction task. Before performing prediction analysis, Expectation Maximization (EM) and Principal Component Analysis (PCA) are employed to address the multi-collinearity problems in the experimental datasets and clustering task, respectively. To the best knowledge of the authors, the combination of prediction methods (ANFIS and SVR), clustering method (EM) and dimensionality reduction (PCA) is applied for the first time in this research in the context of PD diagnosis.

Our study at hand is organized as follows: Section 2 presents related work. Section 3 provides the research methodology along with all approaches used in the proposed model. Section 4 presents the evaluations and finally, conclusions and future work is provided in the Section 5.

## Related Work

For effective diagnosis of Parkinson’s Disease (PD), different types of classification methods were examined by Das^30^. The computation of the performance score of the classifiers was based on various evaluation methods. According to the results of application scores, they found that Neural Networks (NNs) classifier obtains the best result which was 92.9% of accuracy. Bhattacharya and Bhatia^31^ used data mining tool, Weka, to pre-process the dataset on which they used Support Vector Machine (SVM) to distinguish people with PD from the healthy people. They applied LIBSVM to find the best possible accuracy on different kernel values for the experimental dataset. They measured the accuracy of models using Receiver Operating Characteristic (ROC) curve variation. Chen *et al*.^13^ presented a diagnosis PD system by using Fuzzy K-Nearest Neighbor (FKNN). They compared the results of developed FKNN-based system with the results of SVM based approaches. They also employed PCA to further improve the PD diagnosis accuracy. Using a 10-fold cross-validation, the experimental results demonstrated that the FKNN-based system significantly improve the classification accuracy (96.07%) and outperforms SVM-based approaches and other methods in the literature. Ozcift^32^ developed a classification method based on SVM and obtained about 97% accuracy for the prediction of PD progression. Polat^29^ examined the Fuzzy C-Means (FCM) Clustering-based Feature Weighting (FCMFW) for the detection of PD. The author used K-NN classifier for classification purpose and applied it on the experimental dataset with different values of *k*. Åström and Koker^33^ proposed a prediction system that is based on parallel NNs. The output of each NN was evaluated by using a rule-based system for the final decision. The experiments on the proposed method showed that a set of nine parallel NNs yielded an improvement of 8.4% on the prediction of PD compared to a single unique network. Li *et al*.^34^ proposed a fuzzy-based non-linear transformation method to extend classification related information from the original data attribute values for a small data set. Based on the new transformed data set, they applied Principal Component Analysis (PCA) to extract the optimal subset of features and SVM for predicting PD. Guo *et al*.^35^ developed a hybrid system using Expectation Maximization (EM) and Genetic Programming (GP) to construct learning feature functions from the features of voice in PD context. Using projection based learning for meta-cognitive Radial Basis Function Network (PBL-McRBFN), Babu and Suresh (2013) implemented a gene expression based method for the prediction of PD progression. The capabilities of the Random Forest algorithm was tested by Peterek *et al*.^36^ for the prediction of PD progression. A hybrid intelligent system was proposed by Hariharan *et al*.^24^ using clustering (Gaussian mixture model), feature reduction and classification methods. Froelich *et al*.^23^ investigated the diagnosis of PD on the basis of characteristic features of a person’s voice. They classified individual voice samples to a sick or to a healthy person using decision trees. Then they used the threshold-based method for the final diagnosis of a person thorough previously classified voice samples. The value of the threshold determines the minimal number of individual voice samples (indicating the disease) that is required for the reliable diagnosis of a sick person. Using real-world data, the achievement of accuracy of classification was 90%. Eskidere *et al*.^25^ studied the performance of SVM, Least Square SVM (LS-SVM), Multilayer Perceptron NN (MLPNN), and General Regression NN (GRNN) regression methods to remote tracking of PD progression. Results of their study demonstrated that the best accuracy is obtained by LS-SVM in relation to the other three methods, and outperforms latest proposed regression methods published in the literature. In a study by Guo *et al*.^10^ in Central South of Mainland China, sixteen Single-Nucleotide Polymorphisms (SNPs) located in the 8 genes and/or loci (SNCA, LRRK2, MAPT, GBA, HLA-DR, BST1, PARK16, and PARK17) were analysed in a cohort of 1061 PD, and 1066 Normal healthy participants. This study established that Rep1, rs356165, and rs11931074 in SNCA gene, G2385R in LRRK2 gene, rs4698412 in BST1 gene, rs1564282 in PARK17, and L444P in GBA gene have an independent and combined significant effect on PD. As a final point, this study has reported that SNPs in these 4 genes have more pronounced effect on PD.

From the literature on the prediction of PD progression, we found that at the moment there is no implementation of Principal Component Analysis (PCA), Gaussian mixture model with Expectation Maximization (EM) and prediction methods in PD diagnosis. This research accordingly tries to develop an intelligent system for PD diagnosis based on these approaches. Hence, in this paper, we incorporate the robust machine learning techniques and propose a new hybrid intelligent system using PCA, Gaussian mixture model with EM and prediction methods. Overall, in comparison with research efforts found in the literature, in this research:

- A comparative study is conducted between two robust supervised prediction techniques, Adaptive Neuro-Fuzzy Inference System (ANFIS) and Support Vector Regression (SVR).
- EM is used for data clustering. The clustering problem has been addressed in many diseases diagnosis systems^13^,^37^. This reflects its broad appeal and usefulness as one of the steps in exploratory health data analysis. In this study, EM clustering is used as an unsupervised classification method to cluster the data of experimental dataset into similar groups.
- ANFIS and SVR are used for prediction of PD progression.
- PCA is used for dimensionality reduction and dealing with the multi-collinearity problem in the experimental data. This technique has been used in developing in many disease diagnosis systems to eliminate the redundant information in the original health data^27^,^28^,^29^.
- A hybrid intelligent system is proposed using EM, PCA and prediction methods, Adaptive Neuro-Fuzzy Inference System (ANFIS) and Support Vector Regression (SVR) for prediction of PD progression.

## Research Methodology

**Figure 1:**
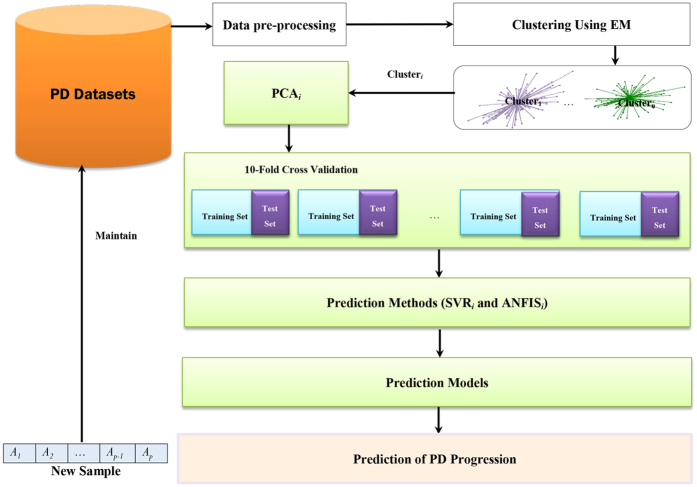
Proposed model for prediction of PD progression.

Clinical decision support systems help healthcare professionals make diagnosis decisions based on patient data. These systems can be developed by machine learning techniques. They can utilize machine learning to learn from past data and recognize patterns. Focusing on the prediction problem of PD progression, the present study uses PCA, EM, SVR and ANFIS methods. These methodologies are addressed in the following sections. The general framework of proposed model is shown in Fig. 1. We propose to rely on ANFIS and SVR to learn the prediction functions. We also uses PCA for dimensionality reduction because the greatest source of difficulties in using prediction methods is the existence of multi-collinearity in many sets of data that in this research PCA will overcome this problem.

### Dataset

The main dataset used for the experiments of this study contains a total of 5875 recordings from 42 subjects that are for 28 men and 14 women (around 200 recordings per patient). The dataset has 16 vocal attributes based on traditional measures (NHR, HNR, shimmer, Jitter) and nonlinear dynamical systems theory (RPDE, DFA, PPE). Each subject of the dataset has been recorded with phonations of the sustained vowel/a/. The dataset’s output is a score on the two outputs of UPDRS, Total-UPDRS and Motor-UPDRS. The ranges of Total-UPDRS and Motor-UPDRS are 0–176 (0 indicating healthy and 176 indicating total disability) and 0–108 (with 0 indicating healthy state and 108 indicating severe motor impairment), respectively. The dataset is available in UCI machine learning repository (Bache and Lichman, 2013). Table 1 presents the 16 features of dataset along with UPDRS scores^25^. The correlation coefficients presented in Table 2 shows that there are strong correlations among the features in PD dataset. These high correlations among the input variables will influence on prediction accuracy of outputs due to the multi-collinearity. Accordingly, to overcome the issue, we apply the PCA on the experimental dataset before performing prediction task.

The second dataset used for method evaluation was obtained from the UCI Machine Learning Repository. The dataset was created by Max Little of the University of Oxford, in collaboration with the National Centre for Voice and Speech, Denver, Colorado, who recorded the speech signals. This dataset contains data from voice recordings of 23 subjects with PD and 8 control subjects. There are a total of 195 recordings, from which 22 different voice measure features have been extracted. Each example also includes a subject identifier and a binary classification attribute which indicates whether or not the subject has PD. The dataset is divided into two classes according to its “Status” column which is set to 0 for healthy subjects and 1 for those with PD. The features of this database are: MDVP:Fo(Hz)(Average vocal fundamental frequency), MDVP:Fhi(Hz) (Maximum vocal fundamental frequency), MDVP:Flo(Hz) (Minimum vocal fundamental frequency), MDVP:Jitter(%), MDVP:Jitter(Abs), MDVP:RAP, MDVP:PPQ, Jitter:DDP (Several measures of variation in fundamental frequency), MDVP:Shimmer, MDVP:Shimmer(dB), Shimmer:APQ3, Shimmer:APQ5, MDVP:APQ, Shimmer:DDA (Several measures of variation in amplitude), NHR, HNR (Two measures of ratio of noise to tonal components in the voice), RPDE, D2 (Two nonlinear dynamical complexity measures), DFA (Signal fractal scaling exponent), Spread1, Spread2, PPE (Three nonlinear measures of fundamental frequency variation), Status (Health status of the subject (one) with Parkinson’s, (zero) is healthy). The dataset is available in UCI machine learning repository.

### EM Clustering

It is well known that the *k*-means clustering is an instance of Expectation Maximization (EM) algorithm which is a general algorithm of density estimation. The EM is a distance based algorithm. Gaussian mixture model with EM algorithm is a powerful approach for clustering. EM algorithm is model based iterative algorithm for solving the clustering problem where the data is incomplete or considered incomplete. EM algorithm is an optimization algorithm for constructing statistical models of the data^38^. In this algorithm each and every data instance belongs to each and every cluster with a certain probability. EM algorithm starts with initial estimates and iterates to find the maximum likelihood estimates for the parameters. The quality of EM algorithm become very good when using huge dataset. It has been also demonstrated that EM is a good clustering method in terms of computation time and accuracy^39^,^40^. In addition, in this study EM is chosen for clustering the data because of its robustness in handling high dimensional and noisy data^41^. The mathematical background of EM algorithm is shown here in this section^38^.

Given a dataset the task of assigning a cluster for each instance in the dataset, is the goal that we aspire for. Let there be *N* data points in the dataset and let us assume that the number of clusters is *k*. Let the index of the cluster be modeled as a random variable and let its probability be given by a multinomial distribution satisfying 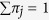, such that


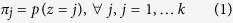


It is assumed that 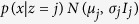 is a Gaussian distribution. *I*_*j*_ denotes the identity matrix of order *j*. The unknown parameters of the model namely the mean *μ*_*j*_, variance 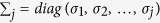 and the distribution function *π*_*j*_ are estimated.


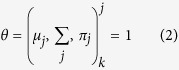



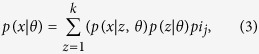


where *z* is an unknown hidden variable. The total log likelihood of all data is given by


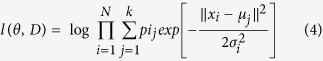


The parameter values that maximize the likelihood function *l*(*θ*, *D*) are the ones that are chosen. Here *D* denotes the data. This optimization is complicated and to solve this some of the unknowns are assumed to be known, while estimating the others and vice versa. For each class, the conditional expectation *z* = *j* of given the data and the parameters.


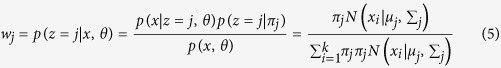


Since each point *x* contributes to *w*_*j*_ in some proportion, for particular *x*_*i*_ we have


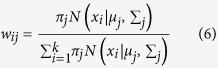


**Figure 2:**
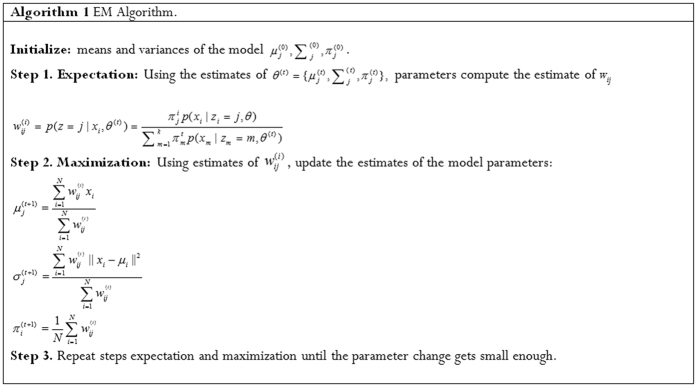
EM algorithm.

The optimization algorithm is called EM and has the following steps. Assume we have some random initial estimates of the means and variances of the model 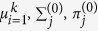. Algorithm 1 in Fig. 2 describes the EM algorithm.

### Principal Component Analysis (PCA)

Medical diagnosis presents an ideal domain for machine learning algorithms. A large part of diagnosis falls under pattern recognition, based on large amounts of data, and machine learning algorithms are well-suited to this task. For an algorithm to be effective in this domain, it needs to be able to handle noisy data, rely on relatively few medical tests, and complement the role of physicians. Principal Component Analysis (PCA) is a tool for data compression and information extraction^27^. There are many correlated or redundant data in process measurements. They must be compressed in a manner to retain the essential information and are easy to display. Also, essential information does not come from an individual process variable but often derives from how the variables change with each other, i.e. how they co-vary. Among the widely used multivariate statistical methods, PCA is an ideal tool for analyzing such data because of its ability to handle large numbers of highly correlated, noisy and redundant variables. Using PCA, a number of related variables are transformed to a set of uncorrelated variables. It is concerned with explaining the variance-covariance structure of a set of variables through a few linear combinations of these variables. Its general objectives are data reduction and interpretation.

### ANFIS

Zadeh first proposed the principles of fuzzy set theory in 1965^42^. With fuzzy sets, transitions between belonging and not belonging are not so abrupt. A Fuzzy Inference System (FIS) is a tool which can be used in a variety of applications such as forecasting. As its name suggests, a FIS uses fuzzy rules and fuzzy reasoning to perform its function^43^,^44^,^45^,^46^. The state of belonging is represented by a membership function. Membership functions (such as Sigmoidal, Triangular, and Gaussian) describe the degree to which a variable belongs to a fuzzy set. It is well-known that FIS are very useful because they allow us to put linguistic information from human experts into computer algorithms. However, a main drawback is the lack of facility to automatically learn from data, which, incidentally is the strength of feed-forward artificial neural networks or ANN. ANFIS, stands for Adaptive-Network-based Fuzzy Inference System or semantically equivalently Adaptive Neuro-Fuzzy Inference System, combines the advantages of FIS and ANN into a single implementation by designing a feed-forward ANN that performs the operations in the FIS. The ANFIS is also a FIS. The ANN training method has also been improved in ANFIS by a hybrid learning scheme. ANFIS uses only the Sugeno-type of fuzzy system with the following constraints. It is a hybrid neuro-fuzzy system proposed by Jang^47^.

**Figure 3:**
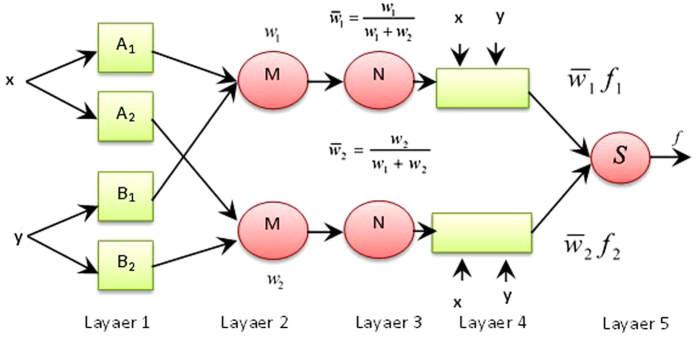
The ANFIS architecture.

Figure 3 shows the ANFIS architecture in five layers. Layer 1 implements fuzzification of crisp input data considering the premise parameters such as membership function parameters. Layer 2 determines the firing strength of a rule by applying T-norm operators on the fuzzy values. Layer 3 normalises the firing strengths produced by Layer 2 while Layer 4 calculates the input for Layer 5 by using the normalised firing strengths and the consequent parameters. Finally, Layer 5 computes the overall output but adding together the outputs of Layer 4. ANFIS uses a hybrid learning algorithm wherein the forward pass employs Least-Squares Estimate (LSE) to identify the consequent parameters while the backward pass uses gradient descent to update the premise parameters.

### SVR

As a powerful machine learning technique, Support Vector Machine (SVM) is becoming increasingly popular. Support Vector Regression (SVR) is an extension of the support vector classifier which estimates the continuous function of certain training data sets^48^. SVR is able to model complex non-linear relationships by using an appropriate kernel function that maps the input matrix *X* onto a higher-dimensional feature space and transforms the non-linear relationships into linear forms. Suppose there is a given training data set with l independent and identical distribution samples,


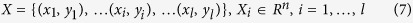


SVR seeks an optimal function 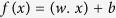 where, *w* is the weight vector and *b* ∈ *R* is the threshold value, and thus minimizes the expected risk of prediction. Introducing the slack variable *ξ*_*i*_ and 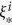, this problem can be described as follows,


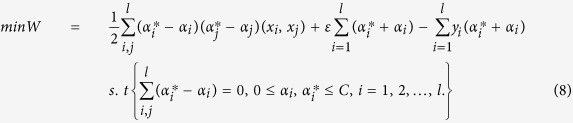


In the case of nonlinearity, samples are mapped into a feature space *H* from input space *R*^*n*^ by the map 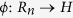. The optimal function is then solved in the feature space *H* to make the predetermined risk function minimization. According to the Mercer theorem, there is a kernel function *K*(0, 0) and it should be met 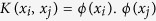. After the introduction of kernel function, the regressive function becomes


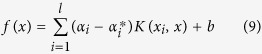


where *α*_*i*_ and 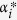 are Lagrangian multipliers.

In this study, suppose the current working set is *X*. Firstly, *X* is clustered by EM; thus, *X* is clustered to 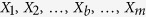 (*b* = 1, …, *M*; *M* is the number of clusters). Then, each *X*_*b*_ is trained by SVR, respectively, and its corresponding training functions *f*(*x*) can be obtained. For each sample (*x*_*c*_, *y*_*c*_) (for prediction), its distance to each cluster is first calculated (Euclidean distance between the observation and the cluster center), and after performing PCA, prediction is carried out using SVR.

### Cross-validation

**Figure 4:**
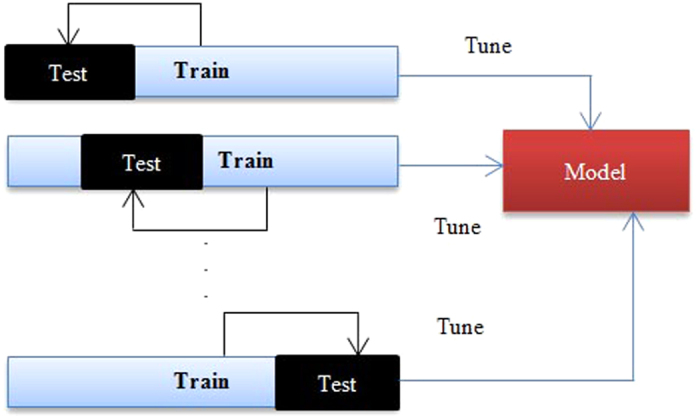
K-fold cross validation.

Cross-validation is a statistical method that in this research is used for the performance evaluation of learning algorithms and performance of a predictive model on an unknown dataset. For this reason, using cross-validation, the datasets used in the research are divided into several equally sized subsets. The learning model is then trained on some subsets known as training sets. After training process, the model is tested on the remaining subsets, known as test sets. According to the number of subsets partitioned, researcher tests *k*-fold cross-validation. For *k*-fold cross-validation, researchers use *k* result of *k*-fold cross-validation. In the experiments of this research, for the training of models, it is considered to test different *k* for *k*-fold cross-validation, so that researchers can make sure that there are enough training instances to learn the models. K-fold cross-validation, as illustrated in Fig. 4, involves partitioning the original sample into randomly partitioned *k* subsamples (as we selected *k* = 10)^49^.

## Results and Discussion

The experimental results of the proposed expert system for the prediction of PD progression are explained in this section. Here, the results of applying all incorporated methods in the proposed system are discussed.

### Clustering with EM algorithm

**Figure 5:**
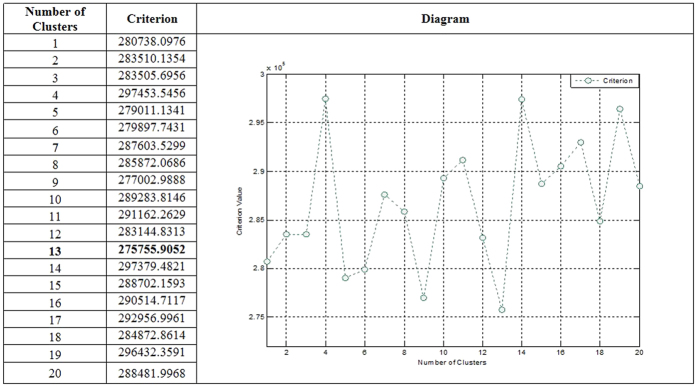
Best cluster using EM algorithm.

**Figure 6:**
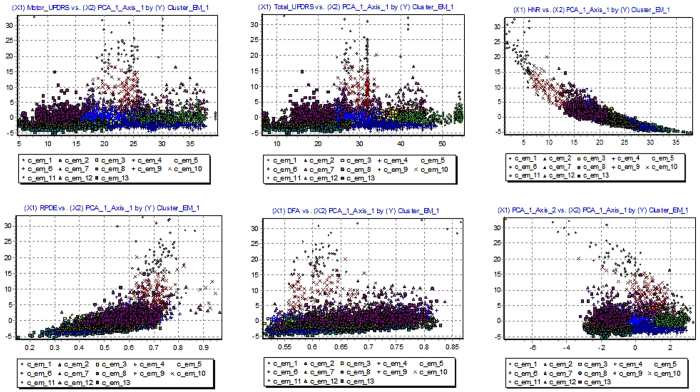
Visualization of clusters.

We applied the EM clustering on PD datasets. In every clustering method, choosing the right number of clusters is important. In EM clustering, with the Gaussian mixture model, the likelihood must be optimized. Hence, for this optimization, the best cluster number is selected by evaluating various values for the number of clusters. It should be noted that according to Pelleg and Moore^50^, we used information theoretic criterion like the Akaike Information Criterion (AIC)^51^ to choose the value optimal number of cluster. Accordingly, in the experimental datasets, we have used a resubstitution AIC estimate and evaluated a number of clusters from 1 to 20. In addition, in the clustering procedure, we applied 10-fold cross validation to obtain unbiased result. In Fig. 5, we present the various numbers of clusters for first dataset to select the best cluster based on chosen criterion. This figure shows that the best criterion value (275755.9052) is obtained when 13 clusters are generated by EM. In Fig. 6, the clusters generated by EM are visualized. For visualizing the dataset clusters into the original space, a PCA is used in order to obtain a 2D representation. It was used to visualize clusters in the scatter plot using the first and second PCs. These clusters are used in PCA and then ANFIS for prediction models. In addition, the cluster centers are used to assign newly arriving data points to a cluster based on their Euclidean distance. It should be noted that for the second dataset, EM generated 3 clusters.

### Solving multi-collinearity issue using PCA

We applied PCA after the initial clustering process individually on each cluster and determined a suitable number of PCs to retain for each cluster. Then, as inputs in ANFIS, we used the PCs for Motor-UPDRS and Total-UPDRS predictions. Following this approach, it allowed us to achieve a high prediction accuracy with lower computation time in predicting the Motor-UPDRS and Total-UPDRS predictions. From the experimental dataset, if we consider input variables in the matrix X, the procedure of dimensionality reduction for overcoming the multi-collinearity can be defined in two steps as follows:

- Perform PCA on matrix *X* that consists of vocal attributes of PD.
- PCs selection from PCA.

**Figure 7:**
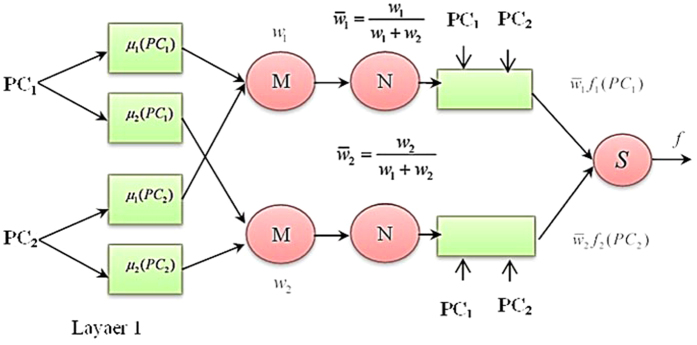
PCA-ANFIS network structure with two PCs.

The selected number of PCs along with the desired output (Motor-UPDRS and Total-UPDRS) are employed in developing the inferential models. Figure 7 illustrates the PCA-ANFIS network structure with two PCs.

### The structure of PCA and ANFIS

**Figure 8:**
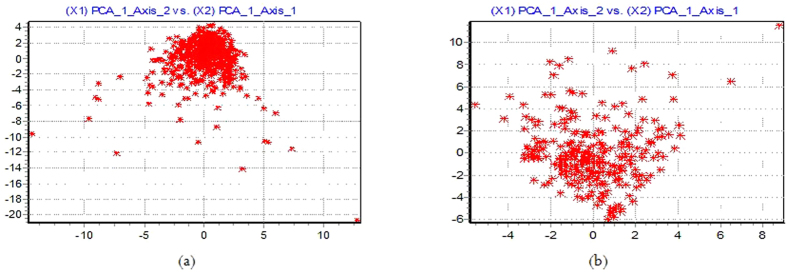
Scatter plots of PCA results for (**a**) cluster 1 and (**b**) cluster 13.

In this study, the main objective of using PCA was to reduce the dimensionality of experimental dataset. Using PCA, we kept as much the useful information in the original datasets by transforming the original input variables to a new set of variables, Principal Components (PCs). The generated PCs by PCA are basically uncorrelated and ordered based on the information provided where the first PC includes most important information provided by the original variables. For constructing a PCA initialization model of PD progression prediction, the PD progression datasets were sufficiently described using some chosen parameters in relation to the original variables with no significant loss of information. In addition, by this way we also could solve the issue of multi-collinearity in the data and accordingly select the number of PCs that sufficiently represented the original data. We applied PCA on the clusters obtained by EM algorithm that the results in the following are presented. It should be noted the results were obtained from datasets without considering the outputs. In PCA, choosing the right number of factors is an important task. If we select too many factors, the noise from the sampling in the analysis will be included. If we choose too few factors, the relevant information will be lost. To overcome this issue, we applied the rule proposed by Cattell^52^ to identify the most important factors in PCA analysis. Using the rule proposed by Cattell^52^, we have selected the most important PCs generated by PCA for each clusters. In Table 3, we have summerized the selected PCs for each cluster of the first dataset. We keep the PCs which provide the significant information and ignore those components with less significance. From the Table 3, it can be found that for Cluster 1 and Cluster 13, nine PCs are selected as they provide significant percentage of information. For Clusters 3, 5, 9 and 11, eight PCs and for Clusters 2, 4, 10 and 12, five PCs are selected. The graphical representations are shown in Fig. 8 which projects the observations in the first two dimensions for Clusters 1 and 13.

### Prediction using ANFIS

We use three set of data for ANFIS modelling which are: training, checking and testing data. The training data is used for constructing the prediction models of ANFIS. The rows of training data are selected as inputs and output for construction the target model. To test generalization capability of the FIS, checking data is then used at each epoch. The checking data also prevents over-fitting and verifies the ANFIS models. Similar to the format of training data, the formats for the checking and testing data are defined data but generally their elements are different from those of the training data. In this study, the fuzzy rule based system was developed through several consequent steps. In the fuzzification step, ANFIS takes the inputs and determine the degree to which they belong to each of the appropriate fuzzy sets via membership functions (Gaussian). After developing membership functions, ANFIS extracted fuzzy rules from the PD datasets to be used in the fuzzy rule based system. Then, in the defuzzification step, the fuzzy outputs are converted into a scalar output quantity, as the output of each rule is fuzzy. It should be noted that as we implemented the fuzzy rule based system in Matlab software, the centroid of area (COA) method was used for deffuzification purpose. COA is the most popular defuzzification method, which returns the center of area under the curve.

**Figure 9:**
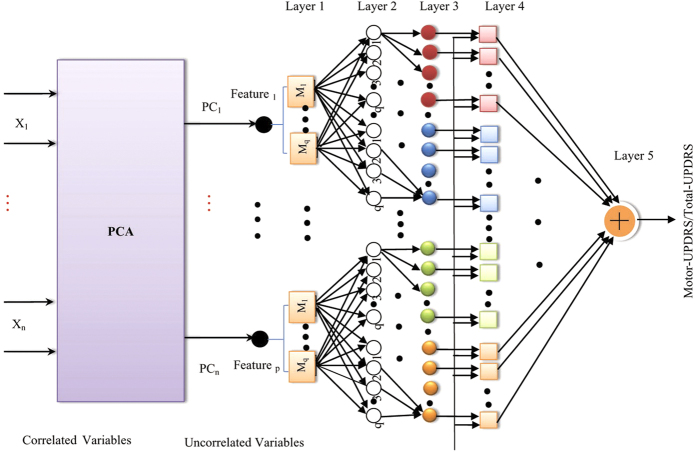
PCA-ANFIS for Predicting Motor-UPDRS/Total-UPDRS.

The results of defuzzification step are then used for the Motor-UPDRS and Total-UPDRS predictions. After applying PCA on clusters, ANFIS models were developed to find the relative importance of criteria and predict the Motor-UPDRS and Total-UPDRS based on input variables. 13 ANFIS models were totally developed based on inputs and output of data for the clusters. Since PCs were selected as inputs of ANFIS models, in the fuzzification steps, for all PCs the degree to which they belong to each of the appropriate fuzzy sets via MFs were determined. Because of its smoothness and concise notation, Gaussian MF is popular method for specifying fuzzy sets. The curves in this type of MF have the advantage of being smooth and nonzero at all points. In addition, this type of MF provided ANFIS models with minimum prediction errors compared to the other types of MFs. Hence, in this paper, we selected Gaussian MF and developed the ANFIS models base on this type of MF. The developed PCA-ANFIS architecture is illustrated in Fig. 9. From this figure, it can be seen that using the PCA approach, dimensionality of PD dataset can be adequately reduced. Also, later, we will demonstrate that PCA overcome multi-collinearity issue in the data and accordingly accuracy improvement in relation to solely using ANFIS.

Tables 4 and 5 present the MFs for 3 PCs generated by PCA-ANFIS for Motor-UPDRS and Total-UPDRS, respectively. From these tables, it can be seen that Gaussian MFs are considered for PCs by three linguistic variables Low, Moderate and High. In Table 4, for each PC, the Gaussian MFs are generated by PCA-ANFIS in three main groups. The range of PC1 for linguistic variable Low, Moderate and High are defined as [3.726 −5.968], [3.719 2.796] and [3.747 11.55], respectively. In Table 5, the range of PC1 for linguistic variable Low, Moderate and High are defined as [3.727 −5.966], [3.717 2.797] and [3.752 11.54], respectively.

**Figure 10:**
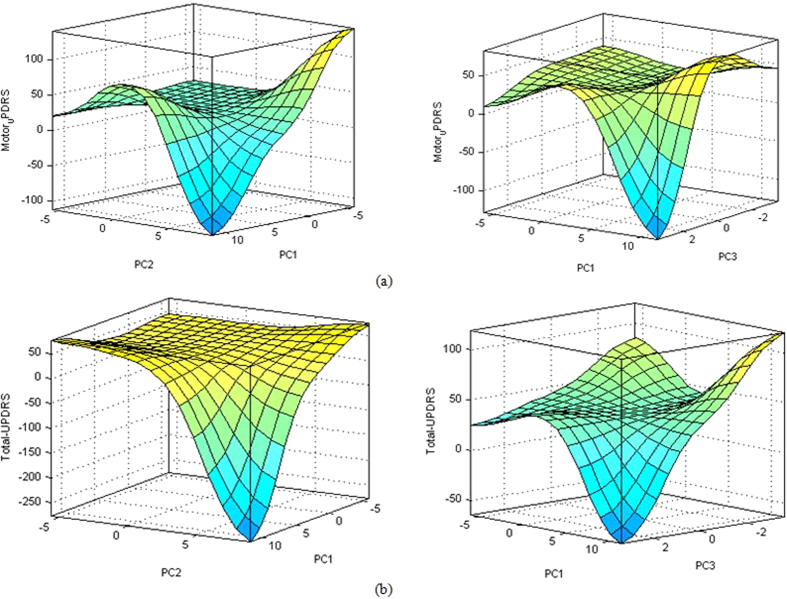
Interdependency of any two PCs and (**a**) Motor-UPDRS and (**b**) Total-UPDRS.

Through control surface, Fig. 10 illustrates the interdependency of four inputs parameters (PCs) and the Motor-UPDRS and Total-UPDRS obtained from the fuzzy rules generated by PCA-ANFIS. The level of Motor-UPDRS and Total-UPDRS can be depicted as a continuous function of its input parameters as PC1, PC2 and PC3. The surface plots depict the variation of Motor-UPDRS and Total-UPDRS based on identified fuzzy rules.

**Figure 11:**
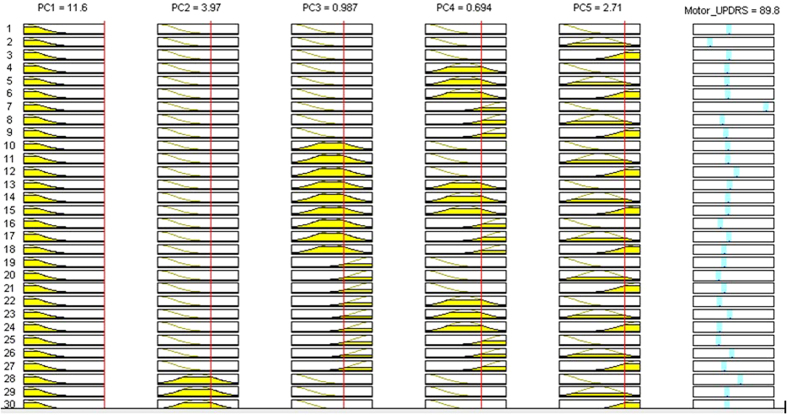
Prediction of Motor-UPDRS based on three PCs in second cluster.

**Figure 12:**
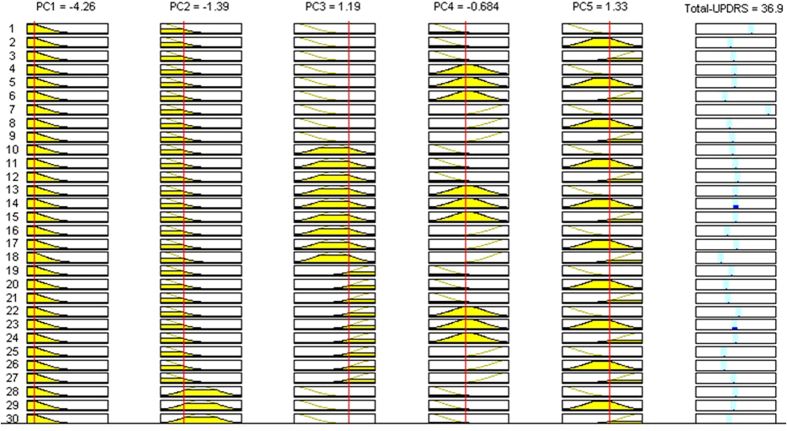
Prediction of Total-UPDRS based on 5 PCs in second cluster.

From the fuzzy rule viewer of established PCA-ANFIS model shown in Figs 11 and 12, the process of Motor-UPDRS and Total-UPDRS prediction by selecting the MFs can be better visualized. From the fuzzy rule viewer in Fig. 11, when the input PC1 is at 11.6, PC2 at 3.97, PC3 at 0.987, PC4 at 0.694, and PC5 at 2.71, an output of Motor-UPDRS at 108 out of 108 is obtained. In addition, from the fuzzy rule viewer in Fig. 12, when the input PC1 is at −4.26, PC2 at −1.39, PC3 at 1.19, PC4 at −0.684, and PC5 at 1.33, an output of Total-UPDRS at 36.9 out of 176 is obtained. It should be noted that COA was used for deffuzification purpose. From Figs 11 and 12, it can be seen that the Motor-UPDRS and Total-UPDRS can be predicted using generated PCs instead of using original variables. Hence, choosing the right number of PCs is important for Motor-UPDRS and Total-UPDRS prediction. As we noted earlier, the eigenvalues that are associated with the factors in each cluster are indicators of their importance and we used those factors as inputs for Motor-UPDRS and Total-UPDRS prediction in ANFIS.

For evaluating the PCA-ANFIS model, two measures of accuracy are used to determine the model capability for predicting the Motor-UPDRS and Total-UPDRS. For this reason, the models are evaluated by two estimators Mean Absolute Error (MAE) and coefficient of determination *R*^2^. The coefficient of determination *R*^2^ provides a value between [0, 1] about the training of the proposed network. A value closer to 1 stands for the success of learning. These estimators are determined by Eqs. 10 and 11.


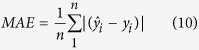



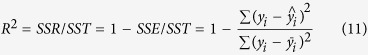


where *n* is the number of observations or samples, 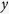 is the observed value, 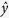 is the predicted value and 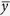 is the average of 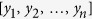.

To experimentally show the effectiveness of clustering, ANFIS and PCA, we perform the experiments on PD dataset. It should be noted that, for ANFIS models, we selected the best configurations in terms of MFs type, type of trainings and number of training. The Gaussian MF type showed the best performance in relation to the Triangular one. In addition, we selected hybrid learning (training) algorithm in ANFIS. This type of learning algorithm combines the least squares estimator and the gradient descent method. Using the hybrid method, the ANFIS models generated fuzzy rules by enumerating all possible combinations of MFs of all original inputs and PCs. Compared with the ANFIS for Motor-UPDRS and Total-UPDRS prediction, the models that used ANFIS with incorporating PCA obtained lower computation time in all models as the computation time for ANFIS is moderately large when the number of inputs is increased (curse of dimensionality)^53^. This can be a main disadvantage of solely using ANFIS for the problem of Motor-UPDRS and Total-UPDRS predictions. Hence, this problem connected to the ANFIS was overcome with incorporating the PCA before applying ANFIS. This incorporation of PCA caused the reduction in number of inputs and accordingly hidden layers, number of MFs and rules. Evidently, the training time of prediction models was significantly reduced.

For error estimation in the clusters of EM, after 200 epochs, the averages MAE and *R*^2^ were calculated (see Table 6). The MAE and *R*^2^ were calculated based on Motor-UPDRS and Total-UPDRS prediction. It should be noted that we used 10-fold cross validation and average test accuracy for each cluster. In Table 7, the computation time (ms) results of PCA-ANFIS are also presented.

### Prediction using SVR

**Figure 13:**
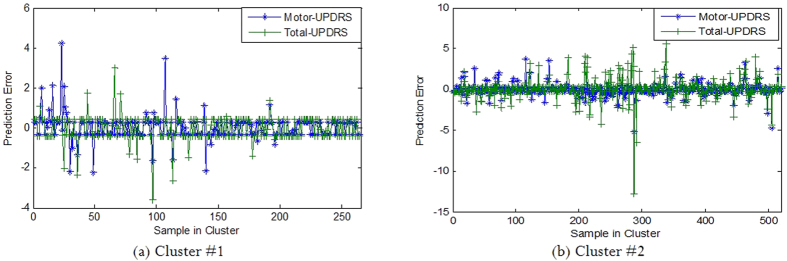
Prediction error of regression approach.

The method used in this study is LIBSVM developed by Chang and Lin^54^. The models of classification were trained under a 4 GHz processor PC and Microsoft Windows 7 running MATLAB 7.10 (R2010a). Results for SVR learner, along with the parameters selected to obtain that result are described below. The results here are based on using all the features. We applied epsilon-SVR approach on the experimental dataset for constructing prediction models. A variety of kernels were used, including linear, 2-, 3-, and 4-degree polynomial, RBF and sigmoid tanh. From the results, we found that RBF kernel is more accurate in prediction task. The C parameter for RBF kernel, which is a trade-off between training error and SVR margin, was set to 0.01. Mean Absolute Error (MAE) and *R*^2^ (coefficient of determination) have been used for performance evaluation of the proposed method for the prediction of PD progression. The error prediction of regression approach on test dataset is visualized in Fig. 13. The plots of this figure demonstrate that the error rate of SVR regression with RBF kernel is relatively low.

From the results of using SVR regression (see Table 8) with RBF kernel, we can see that the regression prediction accuracy is good. In Table 9, the computation time (ms) results of SVR with RBF kernel is also presented. From the results presented in this table, the computation time for Total-UPDRS and Motor-UPDRS are 262035 and 272250, respectively. It should be noted that we applied 10-fold cross validation approach with 10 trials. Data was shufed then divided into 10 sections, and the learner was trained on 9 of those sections and tested on the 10th. The test section was rotated such that this process occurred 10 times. The performance was calculated by averaging the results obtained from each fold for all clusters.

### Comparisons of methods

In the previous sections, we have evaluated the proposed method for Total-UPDRS and Motor-UPDRS predictions. All learners were evaluated using 10-fold cross validation. The dataset for testing the methods contained a total of 5875 recording from 42 subjects, with 16 vocal attributes each. The primary dataset includes these 16 attributes along with 6 additional voice recording features. The dataset’ output was a score on the UPDRS. For comparisons, the performance results of prediction methods that were considered in the experiment with the experimental data are shown in Table 10. The results demonstrate that the accuracies of SVR using RBF kernel are the best on Total-UPDRS and Motor-UPDRS in relation to other methods. Comparison of performance in predicting Motor-UPDRS and Total-UPDRS for PCA-NN and PCA-ANFIS on experimental dataset show that the proposed PCA-ANFIS method is more accurate. However, when compared with PCA-SVR, it can found that prediction errors for PCA-SVR models of EM clusters are lower than other methods (PCA-NN, PCAANFIS, EM-PCA-ANFIS) with high values of coefficient of determination. Hence, in relation to the PCA-ANFIS, our method using EM, PCA and SVR helps to improve the prediction accuracy of Motor-UPDRS and Total-UPDRS by more than 6% and 9% for Motor-UPDRS and Total-UPDRS, respectively. Moreover, it can be found that the accuracy of method which uses prediction techniques with EM and PCA is higher than those methods that use solely NN and ANFIS. These show the effectiveness of incorporating the clustering and PCA techniques for the prediction accuracy of PD progression. The superiority of EM-PCA-ANFIS and EM-PCA-SVR can be explained by the fact that these methods have used clustering and noise removal methods before the prediction of Motor-UPDRS and Total-UPDRS while the other methods solely rely on prediction methods with PCA.

**Figure 14:**
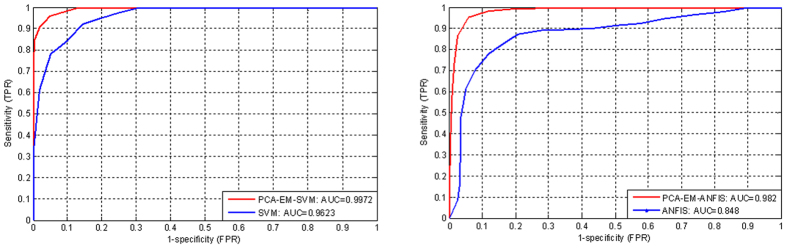
Accuracy of proposed method on the second PD dataset.

To evaluate and show the predictive accuracy of the proposed model on the second PD dataset, the Area Under the Curve (AUC) of Receiver Operating Characteristic (ROC) chart is used. ROC is a graphical display that provides the measure of prediction accuracy of the model using sensitivity and specificity. For predicting events, Sensitivity in ROC is used as a measure of accuracy that is equal to the true positive/total actual positive. For predicting nonevents Specificity is used as a measure of accuracy that is equal to the true negative/total actual negative of a classifier for a range of cutoffs. In Fig. 14, we present the results of clustering, noise removal, prediction techniques for the accuracy improvement of PD on the second dataset. From the results, we can see that proposed method outperforms the methods which do not apply clustering and noise removal methods. In addition, the SVM-based predication method which uses PCA and EM obtained a highest accuracy (AUC = 0.9972) in relation to the ANFIS combined with PCA and EM (AUC = 0.9820). The results show that the difference of accuracy obtained by PCA-EM-SVM and PCA-EM-ANFIS is not significant but the PCA-EM-SVM (using RBF kernel) outperforms the PCA-EM-ANFIS. The results also demonstrated that the method which solely uses ANFIS obtains the lowest accuracy (AUC = 0.8480) compared with the SVM-based predication method (AUC = 0.9623). Compared with the methods in the literature, our proposed method proves to have a better accuracy in relation to the accuracy obtained by Neural Network^30^,^55^, Decision Tree^30^, SVM^55^, Fuzzy C-Means (FCM) Clustering-Based Feature Weighting (FCMFW)^29^ and PCA-FKNN^13^.

The major findings of this study are that the prediction methods are integrated with PCA and EM improved the accuracy prediction of PD. The superiority of the present method can be explained by the fact that our model appropriately combines the noise removal and clustering techniques for predicting PD progression. In addition, the obtained experimental results in this research on a real-world PD dataset demonstrate that tracking PD symptom progression can be effectively predicted by the UPDRS. In summary, the findings of our experiments on public PD datasets show the effectiveness of incorporating the clustering and PCA methods in improving the prediction accuracy of PD progression.

## Conclusion and Future Work

Remote tracking of UPDRS using voice measurements is an effective screening step prior to an appointment with a clinician. Developing computational tools using data mining techniques assists the medical expert to predict Parkinson’s Disease (PD) in the patient faster and recognize the subjects at an early stage. PD is often difficult to diagnosis, but even at early stages, small vocal differences may be machine-detectable. Using this information, it becomes possible to predict PD using voice recordings from potential patients. In this paper, we propose a new hybrid intelligent system for the prediction of PD progression using machine learning techniques. We applied EM clustering algorithm to cluster the experimental PD datasets and prediction methods for prediction of PD progression. In addition, PCA was used for dimensionality reduction and to address multi-collinearity in the datasets. In order to analyze the effectiveness of the proposed method and validate the system, several experiments were conducted using real-word datasets. The datasets were taken from Data Mining Repository of the University of California, Irvine (UCI). The prediction models then were constructed using the features of the experimental datasets, MDVP:Fo(Hz)(Average vocal fundamental frequency), MDVP:Fhi(Hz) (Maximum vocal fundamental frequency), MDVP:Flo(Hz) (Minimum vocal fundamental frequency), MDVP:Jitter(%), MDVP:Jitter(Abs), MDVP:RAP, MDVP:PPQ, Jitter:DDP (Several measures of variation in fundamental frequency), MDVP:Shimmer, MDVP:Shimmer(dB), Shimmer:APQ3, Shimmer:APQ5, MDVP:APQ, Shimmer:DDA (Several measures of variation in amplitude), NHR, HNR (Two measures of ratio of noise to tonal components in the voice), RPDE, D2 (Two nonlinear dynamical complexity measures), DFA (Signal fractal scaling exponent), Spread1, Spread2 and PPE (Three nonlinear measures of fundamental frequency variation). The results showed that for PD datasets the high accuracy can be obtained for PD diagnosis using clustering, noise removal and prediction methods. The results also indicated that the method which combines clustering, PCA and SVR can significantly improve the accuracy of PD prediction. The proposed method can be implemented as an efficient clinical decision support system for PD treatments as it demonstrated that real PD data can accurately predict PD progression. All of the approaches used in this study, may also be applicable to other classification and prediction problems within the medical domain. However, there is still plenty of work in conducting researches on combination of PCA, EM and prediction algorithms for PD disease diagnosis in order to exploit all their potential and usefulness. As we observed from the results obtained by the classical SVR and ANFIS, the method was developed as an off-line method that was trained with a pre-determined PD disease dataset before it can be used for the disease prediction. In addition, the capability of classical SVR and ANFIS was limited by fixed number of training samples in each cluster. Furthermore, although PCA helped to decrease computation time while improving prediction accuracy in both SVR and ANIFS methods, the computation time can be still improved using the incremental version of PCA and prediction methods. In the future work, more attention should be paid to the datasets for PD disease prediction using the SVR, as it outperformed other methods, and especially incremental SVR to reduce the computation time. Hence, in our future study, we plan to develop methods for incremental learning and evaluate them on large datasets to show the effectiveness of the proposed method.

## Additional Information

**How to cite this article**: Nilashi, M. *et al*. Accuracy Improvement for Predicting Parkinson’s Disease Progression. *Sci. Rep.* **6**, 34181; doi: 10.1038/srep34181 (2016).

## Figures and Tables

**Table 1:** Description of the features and UPDRS scores of the first Parkinson’s telemonitoring dataset.

Description	Label	Feature label	Min	Max	Mean	SD
Clinician’s motor UPDRS score, linearly interpolated	Motor-UPDRS	Motor-UPDRS (baseline)	6	36	19.42	8.12
		Motor-UPDRS (after three months)	6	38	21.69	9.18
		Motor-UPDRS (after six months)	5	41	29.57	9.17
Clinician’s total UPDRS score, linearly interpolated	Total-UPDRS	Total-UPDRS (baseline)	8	54	26.39	10.8
		Total-UPDRS (after three months)	7	55	29.36	11.82
		Total-UPDRS (after six months)	7	54	29.57	11.92
Several measures of variation in fundamental frequency	F1	MDVP:Jitter (%)	8E-4	0.1	0.006	0.006
	F2	MDVP:Jitter (Abs)	2E-6	4E-4	4E-5	3E-5
	F3	MDVP:Jitter:RAP	3E-4	0.057	0.003	0.003
	F4	MDVP:Jitter:PPQ5	4E-4	0.069	0.003	0.004
	F5	Jitter:DDP	10E-4	0.173	0.009	0.009
Several measures of variation in amplitude	F6	MDVP:Shimmer	0.003	0.269	0.034	0.026
	F7	MDVP:Shimmer (dB)	0.026	2.107	0.311	0.230
	F8	Shimmer:APQ3	0.002	0.163	0.017	0.013
	F9	Shimmer:APQ5	0.002	0.167	0.020	0.017
	F10	Shimmer:APQ11	0.003	0.276	0.028	0.020
	F11	Shimmer:DDA	0.005	0.488	0.052	0.040
Two measures of ratio of noise to tonal components in the voice	F12	NHR	3E-4	0.749	0.032	0.060
	F13	HNR	1.659	37.875	21.679	4.291
A nonlinear dynamical complexity measure	F14	RPDE	0.151	0.966	0.541	0.101
Signal fractal scaling exponent	F15	DFA	0.514	0.866	0.653	0.071
A nonlinear measure of fundamental frequency variation	F16	PPE	0.022	0.732	0.220	0.092

**Table 2:** Correlation coefficients between the features of PD dataset.

	F1	F2	F3	F4	F5	F6	F7	F8	F9	F10	F11	F12	F13	F14	F15	F16
F1	1															
F2	0.87	1														
F3	0.98	0.84	1													
F4	0.97	0.79	0.95	1												
F5	0.98	0.84	1.00	0.95	1											
F6	0.71	0.65	0.68	0.73	0.68	1										
F7	0.72	0.66	0.69	0.73	0.69	0.99	1									
F8	0.66	0.62	0.65	0.68	0.65	0.98	0.97	1								
F9	0.69	0.62	0.66	0.73	0.66	0.98	0.98	0.96	1							
F10	0.65	0.59	0.60	0.67	0.60	0.94	0.94	0.89	0.94	1						
F11	0.66	0.62	0.65	0.68	0.65	0.98	0.97	1.00	0.96	0.89	1					
F12	0.83	0.70	0.79	0.86	0.79	0.80	0.80	0.73	0.80	0.71	0.73	1				
F13	−0.68	−0.71	−0.64	−0.66	−0.64	−0.80	−0.80	−0.78	−0.79	−0.78	−0.78	−0.68	1			
F14	0.43	0.55	0.38	0.38	0.38	0.47	0.47	0.44	0.45	0.48	0.44	0.42	−0.66	1		
F15	0.23	0.35	0.21	0.18	0.21	0.13	0.13	0.13	0.13	0.18	0.13	−0.02	−0.29	0.19	1	
F16	0.72	0.79	0.67	0.66	0.67	0.62	0.64	0.58	0.59	0.62	0.58	0.56	−0.76	0.57	0.39	1

**Table 3:** Result of PCA on 13 clusters.

Cluster No.	PC1	PC2	PC3	PC4	PC5	PC6	PC7	PC8	PC9	PC10	PC11	PC12	PC13	PC14	PC15	PC16
Cluster 1	✓	✓	✓	✓	✓	✓	✓	✓	✓							
Cluster 2	✓	✓	✓	✓	✓											
Cluster 3	✓	✓	✓	✓	✓	✓	✓	✓								
Cluster 4	✓	✓	✓	✓	✓											
Cluster 5	✓	✓	✓	✓	✓	✓	✓	✓								
Cluster 6	✓	✓	✓	✓	✓	✓										
Cluster 7	✓	✓	✓	✓	✓	✓	✓									
Cluster 8	✓	✓	✓	✓	✓	✓	✓									
Cluster 9	✓	✓	✓	✓	✓	✓	✓	✓								
Cluster 10	✓	✓	✓	✓	✓											
Cluster 11	✓	✓	✓	✓	✓	✓	✓	✓								
Cluster 12	✓	✓	✓	✓	✓											
Cluster 13	✓	✓	✓	✓	✓	✓	✓	✓	✓							

**Table 4:** The information of MFs for second cluster in predicting Motor-UPDRS.

Variables	MF Type	Low	Moderate	High
PC1	Gaussian	[3.726 −5.968]	[3.719 2.796]	[3.747 11.55]
PC2	Gaussian	[3.056 −5.591]	[3.045 1.602]	[3.063 8.784]
PC3	Gaussian	[1.529 −3.545]	[1.472 −0.08309]	[1.471 3.412]

**Table 5:** The information of MFs for second cluster in predicting Total-UPDRS.

Variables	MF Type	Low	Moderate	High
PC1	Gaussian	[3.727 −5.966]	[3.717 2.797]	[3.752 11.54]
PC2	Gaussian	[3.059 −5.587]	[3.04 1.606]	[3.071 8.78]
PC3	Gaussian	[1.537 −3.537]	[1.464 −0.0799]	[1.48 3.405]

**Table 6:** MAE and *R* ^2^ for PCA-ANFIS modelling of predicting Motor-UPDRS and Total-UPDRS using ANFIS.

Method	Measure	MAE	R2
EM-PCA-ANFIS	Motor-UPDRS	0.585	0.887
	Total-UPDRS	0.532	0.923

**Table 7:** Computation time (ms) for Total-UPDRS and Motor-UPDRS using ANFIS.

Cluster No.	Total-UPDRS	Motor-UPDRS
1	68000	87000
2	82000	98000
3	61000	76000
4	91000	103000
5	69000	85000
6	82000	100000
7	110000	147000
8	149000	172000
9	141000	177000
10	105000	139000
11	180000	224000
12	169000	211000
13	82000	120000
Computation Time (ms)	1389000	1739000

**Table 8:** Prediction accuracy for Total-UPDRS and Motor-UPDRS using SVR.

Cluster No.	Total-UPDRS	Motor-UPDRS
1	0.4387	0.4134
2	0.3866	0.4322
3	0.4125	0.4533
4	0.3856	0.3223
5	0.4865	0.4334
6	0.4725	0.5433
7	0.4287	0.4566
8	0.4237	0.4564
9	0.4256	0.4554
10	0.4553	0.5654
11	0.4693	0.5433
12	0.4772	0.5453
13	0.4983	0.5182
Average Accuracy	0.4431	0.4721

**Table 9:** Computation time (ms) for Total-UPDRS and Motor-UPDRS using SVR.

Cluster No.	Total-UPDRS	Motor-UPDRS
1	12250	13650
2	15425	14550
3	10490	11250
4	17045	16200
5	12535	13650
6	15325	16350
7	20490	22200
8	28835	27450
9	26985	28000
10	19450	21650
11	34935	36000
12	32490	33650
13	15780	17650
Computation Time (ms)	262035	272250

**Table 10:** MAE and *R* ^2^ for PCA-ANFIS modelling of predicting Motor-UPDRS and Total-UPDRS.

Method	Measure	MAE	R2
PCA-NN	Motor-UPDRS	0.861	0.721
	Total-UPDRS	0.841	0.745
PCA-ANFIS	Motor-UPDRS	0.662	0.791
	Total-UPDRS	0.634	0.812
PCA-SVR	Motor-UPDRS	0.611	0.825
	Total-UPDRS	0.599	0.831
EM-PCA-ANFIS	Motor-UPDRS	0.585	0.887
	Total-UPDRS	0.532	0.923
EM-PCA-SVR	Motor-UPDRS	0.4721	0.977
	Total-UPDRS	0.4431	0.991
